# Fractionation of AquaSolv Omni Biorefinery Lignins and Their Application in Antioxidant and Ultraviolet‐Protective Films

**DOI:** 10.1002/cssc.202501985

**Published:** 2026-02-18

**Authors:** Daryna Diment, MiJung Cho, Davide Rigo, Michael Hummel

**Affiliations:** ^1^ Department of Bioproducts and Biosystems School of Chemical Engineering Aalto University Espoo Finland

**Keywords:** antioxidant activity, lignin, lignin–carbohydrate complex, post‐biorefinery fractionation, ultraviolet protection

## Abstract

Lignin and lignin–carbohydrate complexes (LCCs) were isolated using AquaSolv Omni (AqSO). The process involves hydrothermal treatment of wood followed by organic solvent extraction, using a sequential fractionation approach with water‐based alkaline (1 wt%), ethanol, and acetone solutions. This strategy allowed for isolating different lignin/LCCs fractions with varying structure and properties. The sequential fractionation approach provided fractions with wide variability of glass transition temperatures ranging from 94°C to 153°C, while demonstrating excellent antioxidant activity with a normalized radical scavenging index up to 13.2 mmol g^−1^. The incorporation of different lignin/LCCs with high antioxidant activity into lignocellulosic film formulation at 1.5 wt%, in which lignin and cellulose were the sole components, demonstrated superior effectiveness in blocking over 90% of ultraviolet (UV) rays (sun protection factor = 6–12), yet maintaining a high transparency of the resulting film. This study underscores the versatility of lignin and its high potential for integration into applications where strong UV and antioxidant protection are concerned without posing any environmental concern.

## Graphical abstract

Sequential fractionation of AquaSolv Omni biorefinery lignins allowed to obtaining of lignin/lignin–carbohydrate complexes with potent antioxidant and Ultraviolet‐shielding properties, showing adaptable glass transition behavior that can be tailored according to the selected application requirements.

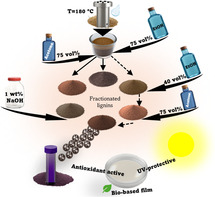

## 1 Introduction

Rapid industrialization has led to a drastic increase in the consumption of nonrenewable fossil fuels, with plastics production significantly contributing to rising CO_2_ emissions in the atmosphere. Consequently, this has a major impact on climate change by increasing atmospheric CO_2_ concentrations, which results in global warming and natural catastrophes [1, 2]. To mitigate the severe consequences of climate change and prevent its further progression, transitioning to renewable sources with a reduced environmental footprint is of paramount importance. Plant biomass is the most abundant and accessible source of renewable chemicals [3, 4], while lignocellulosic biomass has emerged as a carbon‐neutral feedstock. To facilitate its utilization, numerous fractionation strategies have been reported in the literature to enable efficient biomass fractionation, including acid and alkaline treatments [5, 6], supercritical water (scH_2_O) treatment [7, 8], as well as enzymatic hydrolysis [9]. While all these methods have substantial drawbacks, hydrothermal approaches or hydrothermal treatment (HTT), including hot water extraction [4, 10, 11], offer a benign alternative to achieve effective separation of lignin from carbohydrate‐rich fractions.

Lignin/lignin–carbohydrate complexes (LCCs) properties and, thus, ultimate performance are highly dependent on its structure, which originates from lignin origin (softwood or hardwood), processing method (Kraft, lignosulfonates, organosolv, etc.), and isolation method [3]. The use of different solvents for post‐HTT fractionation enables the isolation of lignin/LCCs with distinct properties. By adjusting the extraction parameters, these properties can be tailored to meet the specific requirements of various end‐use applications. As a result, incorporating lignin fractionation into biorefinery concepts could improve waste management sustainability by enabling the production of more homogeneous fractions and higher value‐added lignin‐based substrates and products [12]. Lignin fractionation approaches can be classified according to their underlying principles, with precipitation, extraction, and gradient elution representing the core strategies. Among these, organic solvent extraction is one of the most widely reported methods for lignin fractionation, while solvent‐based sequential extraction has been proven to yield less‐dispersed lignin fractions. The principle of solvent fractionation relies on the partial solubility of polymers in different solvents. Owing to the heterogeneity of lignin, variations in solubility along its structure provide selective dissolution in organic solvents such as ethanol, methanol, dichloromethane, and acetone, yielding fractions with reduced polydispersity. In general, the efficiency of lignin fractionation correlates with the hydrogen‐bonding capacity of the solvent, whereby solvents with higher hydrogen‐bonding capacity preferentially extract higher molecular weight lignin fractions [20, 21]. For instance, sequential solvent extraction (e.g., methanol, acetone, ethyl acetate, petroleum ether) allowed to obtaining of lignin fractions of distinct molecular weights and dispersities [22]. Other approaches, such as solvent‐shifting methods use water‐containing mixtures of organic solvents (e.g., acetone, ethanol, propylene glycol monomethyl ether) to precipitate high‐purity fractions by controlled water addition [23, 24, 25].

While extensive research has focused on various biomass fractionation techniques, relatively few studies have specifically addressed the direct isolation of lignin or LCCs via alkali or solvent extraction following HTT [11]. One of the earliest contributions in this area was by Lora and Wayman, who achieved lignin yields of up to 91.6% using dioxane/water mixtures to extract lignin from hydrothermally treated hardwoods at 175°C–220°C and a liquid‐to‐solid (L:S) ratio of 1 [26]. Similarly, lignin yields ranging from 26% to 50% were reported using the same solvent system on steam‐exploded aspen wood. Chen and Wang isolated four distinct lignin fractions from steam‐exploded stalks through a combination of alkali extraction and subsequent ethanol–water dissolution [27]. Sun et al. reported lignin yields of approximately 50% from eucalyptus fiber pretreated via hydrothermal methods, followed by subsequent extraction using aqueous NaOH solution [28]. These studies highlight the growing interest in selective fractionation techniques following hydrothermal pretreatment, particularly for the recovery of lignin/LCC fractions. In addition, we have recently reported an AI‐optimized method to extract lignins with tailor‐made properties using 75 vol% acetone (aq.) [29] through a green and flexible HHT‐based biorefinery process AqSO Omni (AqSO) [4, 11].

Lignin demonstrates potent ultraviolet (UV)‐blocking properties due to the presence of different chromophores in its structure that include conjugated carbonyls, aromatic rings, and carbon–carbon double bonds, while absorbing a broad spectrum of UV light in the range 250–400 nm [30]. It implies its significance in sunscreen formulations, UV‐protecting films, paints and coatings, car windshields, textiles, adhesives, sealants, and optical materials [31]. To mitigate the damaging effect of UVA (320–400 nm) and UVB (280–320 nm) irradiation, the incorporation of potent UV absorbers is highly requested. UV absorbers are usually represented by minerals and synthetic chemicals. However, an extended exposure to the latter is linked with potential health risks, toxicity to marine life, and long‐term environmental accumulation. Moreover, conventional synthetic antioxidants and UV‐filters, widely used for example in sunscreens, pose severe risks to marine ecosystems, while their toxicity has led to restrictions in sensitive regions such as Hawaii on the sale of oxybenzone‐ and octinoxate‐containing sunscreens to protect coral reefs [16]. On top of that, a vast majority of synthetic UV absorbers are temperature sensitive, which limits their use in polymer processing and composite materials production [17].

One of the most promising strategies to design sustainable yet effective UV absorbers is basedon the targeted biomass processing. To provide both UV and antioxidant protection, lignin is a first‐choice option. This dual functionality is of high importance for thermoplastics production, coatings, and cosmetics as it provides simultaneous photodegradation and oxidation protection [30]. Several works reported enhanced properties after lignin incorporation in different film formulations. For instance, Li et al. achieved enhanced UV blocking ability and ca. 30% higher tensile strength and elongation at break by using lignin as a filler for polybutylene terephthalate films [19]. Simultaneously, lignin‐containing cellulose nanofibers (CNF) films demonstrated higher UV light absorption with remarkable antioxidant and oxygen barrier properties [32, 33]. Other authors reported the improvement in thermal stability and UV‐shielding ability of PVA films after the incorporation of lignin nanoparticles, while possessing high mechanical and antioxidant properties [34, 35, 36].

In the study at hand, a systematic post‐HTT fractionation trials were performed within the framework of the AqSO biorefinery concept, combining different processing conditions (e.g., temperature and *P*‐factor) along with varying fractionation agents, such as 75 vol% acetone (aq.), 75 vol% and 40 vol% ethanol (aq.), and 1 wt% NaOH (aq.). The effects of process parameters and extraction agent (lignin partial solubility) on the resulting structure and properties of the obtained LCCs were evaluated. To provide a better insight into its applicability, fractionated lignins/LCCs were incorporated into the lignocellulosic films, and their UV‐absorbance was tested. The goals of the study at hand are: (i) to identify processing and extraction conditions that yield LCCs with significant thermal, antioxidant, and UV‐shielding properties; (ii) to investigate these properties as performance‐decisive hallmarks relevant to high‐value applications through the production of regenerated lignocellulosic films, and the effect of the selected properties on its UV‐protecting capabilities; and (iii) the development of the process‐structure‐property‐performance correlation for the produced lignins.

## 2 Materials and Chemicals

Acetone (C_3_H_6_O, 95 vol%), deuterated dimethyl sulfoxide (DMSO‐d_6_), ethanol (EtOH) (all analytical grade), 2,2‐diphenyl‐1‐picrylhydrazyl (DPPH), 1‐ethyl‐3‐methylimidazolium chloride ([emim]Cl), sodium hydroxide (NaOH), and microcrystalline cellulose were purchased from Sigma–Aldrich. For the HTT process, extractive‐free birch (*Betula pendula*) sawdust was utilized as the feedstock. A birch wood (*Betula* sp.) stem was further debarked, chipped, and ground into sawdust using a Wiley Mill M02 grinder (0.5 mm average particle size). Following that, the sawdust was screened using a Retsch AS 300 Control Vibratory Sieve Shaker – RAMI to obtain a particle size fraction of 0.5–0.15 mm, which was subsequently exposed to air drying. At the last stage, the obtained sawdust was exposed to acetone (99.9% purity) extraction in a Soxhlet apparatus, aiming to remove the lipophilic extractives and additionally prevent erroneous evaluation of the lignin yield and the resulting structure.

### 2.1 AqSO Process and Sequential Fractionation

**FIGURE 1:**
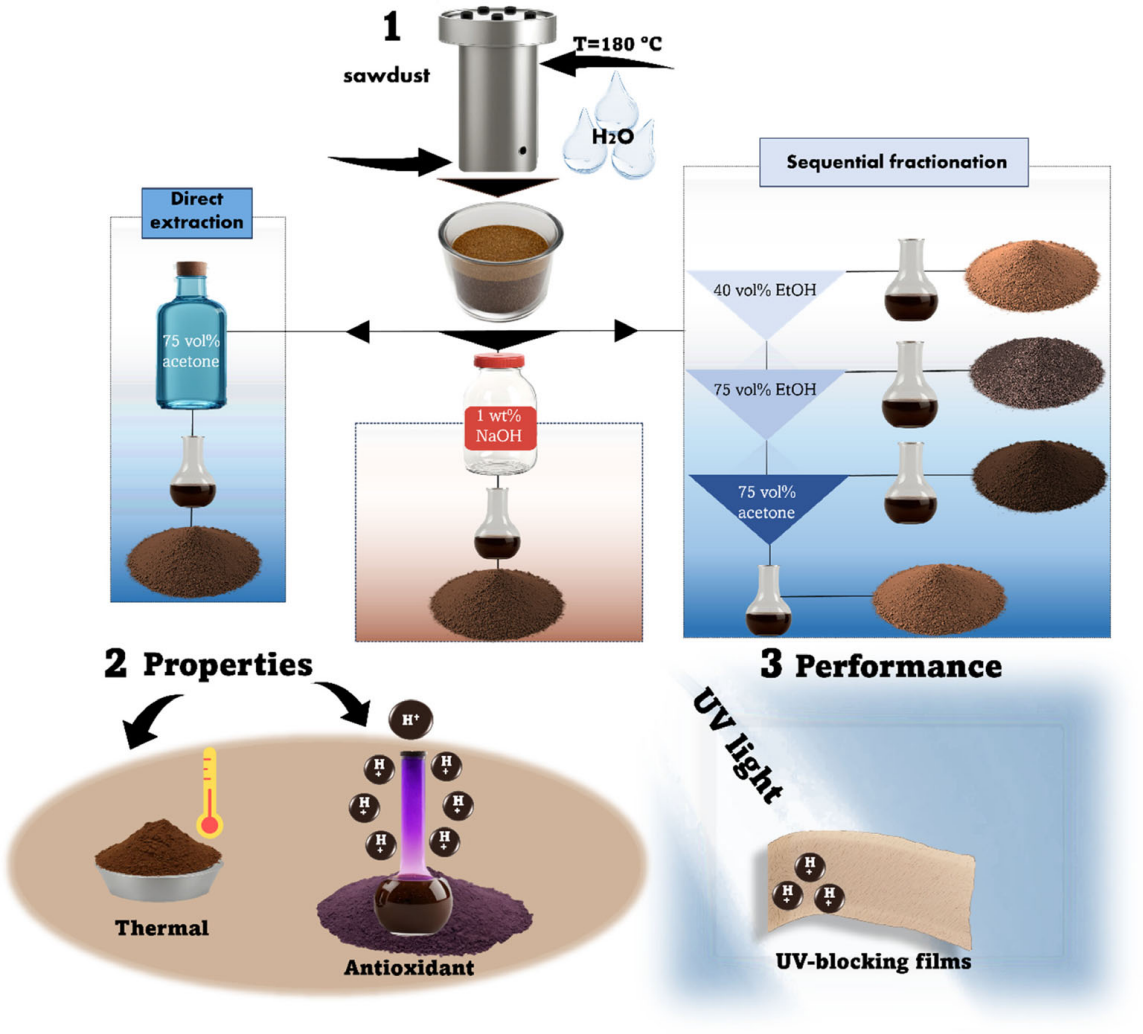
A schematic representation of the workflow implemented in the current work, where (1) At the first stage, the HTT process was performed by loading the chosen amount of sawdust and water in a 1:1 ratio at *T* = 180°C into the reactor. Following that, the obtained treated solids were exposed to: (i) direct extraction with only 75 vol% acetone (aq.) (E1, Table 1); (ii) fractionation with 1 wt% NaOH (aq.) (E6, Table 1); (iii) sequential fractionation (E2, Table 1) with 40 vol% EtOH (aq.) (E3, Table 1), followed by 75 vol% EtOH (aq.) (E4, Table 1), and by 75 vol% acetone (aq.) (E5, Table 1). Lignin solutions obtained during E2, E3, and E4 were separately collected for further isolation as well. The process ultimately yielded six fractionated lignin/LCCs. (2) The antioxidant activity and glass transition temperatures of the isolated lignins were evaluated, and (3) after which they were incorporated as active ingredients in the preparation of UV‐blocking films.

The AqSO process was performed according to the procedure reported by Tarasov et al. and Schlee et al. [4, 11]. The process employed a stainless steel autoclave (inner volume of 120 cm^3^), equipped with temperature control in both the heating block and inside the reactor. It was used to perform a HTT of the extractive‐free sawdust. For quantification and control purposes, the severity of the HTT reaction was expressed through a prehydrolysis intensity factor (*P*‐factor). It converts time and temperature into a single variable that is dependent on the heating curve of the reactor. In other words, the *P*‐factor quantifies the rate of pre‐hydrolysis. While the relative reaction rate reflects the reaction rate at a given temperature relative to the reference reaction rate at 100°C (Equation (1)) [13]:

(1) $$\mathrm{Ln}\frac{k_{H,\left(T\right)}}{k_{100^{{}^{\circ}}C}}=\frac{E_{A,H}}{375.15R}-\frac{E_{A,H}}{RT}$$

where $k_{H,(T)}$ is the rate constant of xylan hydrolysis at the chosen temperature *T*, and *E*_A,H_ is the activation energy. In light of this, *P*‐factor calculations are dependent on the activation energy of the fast‐reacting xylan. The activation energy of 125.6 kJ mol^−1^ was taken into account for the fast‐reacting xylan hydrolysis. Subsequently, the *P*‐factor for each experiment was calculated according to (Equation (2)) [13]:

(2) $$\begin{array}{c} P-\text{factor}=\int_{0}^{t_{f}}\frac{k\left(T(t)\right)}{k_{100{}^{\circ}C}}dt=\int_{0}^{t_{f}}e^{40.48-\frac{15106}{T(t)}}dt \end{array}$$

where *k* is the rate constant of the reaction, *T* is the reactor temperature (in Kelvin), and *t*_f_ is the residence time (in hours). The reactor temperature was set to 180°C with a liquid‐to‐solid ratio of 1 for all performed experiments. *P*‐factors were chosen in the range of 500–2000. After HTT, the treated solids were exhaustively washed with deionized water (50 mL), and then thoroughly extracted with different solvents: (i) only 75 vol% acetone (aq.); (ii) sequentially extracted with 40 and 75 vol% EtOH (aq.), followed by 75 vol% acetone (aq.); (iii) and only 1 wt% NaOH (aq.) to obtain LCC‐containing fractionated lignins (FLs) (Figure 1). Thereafter, the resulting FLs were isolated using rotary evaporation (*T* = 40°C, *p* = 20 mbar), followed by vacuum oven drying until a constant weight was reached at 40°C and 5 mbar with the P_2_O_5_ as the drying agent. However, in the case of 1 wt% NaOH (aq.), the resulting FL was isolated by H_2_SO_4_ precipitation with a subsequent centrifugation. All experiments were performed three times. The FL samples were labeled according to the convention: *P*‐factor‐fractionation agent (e.g., P2000‐A75) represented in Table 1.

**TABLE 1:** Extraction conditions employed in the current work.

Extraction	Label
Only 75 vol% acetone (aq.)	E1
40 → 75 vol% EtOH (aq.) → 75 vol% acetone (aq.)	E2
40 vol% EtOH (aq.)	E3 (isolated from E2)
75 vol% EtOH (aq.)	E4 (isolated from E2)
75 vol% acetone (aq.)	E5 (isolated from E2)
1 wt% NaOH (aq.)	E6

### 2.2 Nuclear Magnetic Resonance (NMR) Spectroscopy

#### 2.2.1 ^1^H‐^13^C Heteronuclear Single Quantum Coherence Spectroscopy (HSQC)2D HSQC

NMR spectra were recorded on a Bruker AVANCE 600 NMR spectrometer equipped with a CryoProbe. Concisely, about 80 mg of each FL sample was dissolved in 0.6 mL of DMSO‐d_6_. An acquisition time was set as 77.8 ms for the ^1^H‐dimension, and 36 scans per block were collected using 1024 complex data points. For the ^13^C‐dimension, the acquisition time was set to 3.94 ms, and 256 time increments were recorded. Following that, the 2D HSQC NMR data were processed with 1024 × 1024 data points, applying the Qsine function for both the ^1^H and ^13^C dimensions. The DMSO peak at *δ*_C_/*δ*_H_ 39.5/2.49 ppm/ppm was used to calibrate the chemical shifts. The quantity of different lignin and LCC signals was normalized and expressed as the intensities of the signals in mol% (per 100 aromatic units (Ar)), assuming that: *G* + *S* = *G*_2 _+ *S*_2,6_/_2_ = 100 Ar [4, 11]. The sum of the signals detected at the *G*_2_ position of guaiacyl units and at the *S*_2_ and *S*_6_ (*S*_2,6_) positions of syringyl units implies that the condensation at these positions is negligible. The corresponding signals of the areas of interest were assigned according to previously reported works [4, 11, 37, 38, 39]. The processed 2D HSQC data were semi‐quantified by exploring the coefficient (k), which complies with the condition: *k*(*G*_2_ + *S*_2,6_/_2_) = 100. Following that, this coefficient was applied to values corresponding to the intensity of the signals of the areas of interest.

#### 2.2.2 ^31^P NMR

Determination, assignment, and subsequent quantification of different hydroxyl groups were carried out using ^31^P NMR according to our optimized method [40]. The spectra were recorded on a Bruker NMR Spectrometer AV III 400 with an acquisition time set to 1 s, relaxation delay of 5 s, and number of scans set to 128. In short, ca. 40 mg of each lignin sample was dissolved in 0.4 mL of a freshly prepared mixture of pyridine and CDCl_3_ (1.6 : 1, v/v). Following that, 100 μL of e‐HNDI solution (0.3 μmol mg^−1^) as an internal standard (IS) and 50 μL of chromium(III) acetylacetonate solution (11.4 mg mL^−1^) were added. Finally, 100 μL of derivatization agent (TMDP) was added, and the vial was vortexed until a homogeneous solution was obtained, which was transferred into an NMR tube. The resulting spectra were phased and calibrated according to the signal of the 2,2′‐oxybis(4,4,5,5‐tetramethyl‐1,3,2‐dioxaphospholane) water‐derivatized product at 132.2 ppm. A linear function was employed to provide baseline correction.

### 2.3 Molar Mass Distribution (MMD)

High‐performance liquid chromatography was performed using an Agilent 1100 system. Calibration was carried out using polystyrene sulfonate standards (molecular weights ranging from 1000 to 64000 g mol^−1^), ascorbic acid (176 g mol^−1^), and sodium chloride (58 g mol^−1^), with detection via a refractive index detector. MMD was based on UV detection at 280 nm. Separation was achieved using three Polymer Standards Service MCX columns (300 × 8 mm) with pore sizes of 100, 500, and 1000 Å. The mobile phase was 0.1 M NaOH, applied at a flow rate of 0.7 mL min^−1^. An injection volume of 50 μL was used, and the sample concentration was set at 2 mg mL^−1^ in the eluent. Chromatograms are provided in the ESI (Electronic Supporting Information).

### 2.4 Glass Transition Temperature

Glass transition temperatures (*T*_g_) of the resulting FLs were determined using differential scanning calorimetry (DSC). Measurements were conducted on a Discovery DSC 250 instrument (TA Instruments, USA). Approximately 7 mg of each sample was analyzed under a nitrogen flow of 50 mL min^−1^. The samples were first heated from 25°C to 200°C at 10°C min^−1^, followed by a 5 min isothermal hold, and cooled to 25°C at the same rate, while a second heating ranged from 25°C to 220°C at 10°C min^−1^. *T*_g_ values were further analyzed based on the second heating cycle using TA Universal Analysis software. The resulting DSC curves are provided in the ESI.

### 2.5 Antioxidant Properties

Antioxidant activity of FLs was determined according to the recently suggested approach [41], while using 2,2‐diphenyl‐1‐picrylhydrazyl (DPPH) as a reactive free radical [14, 15, 18]. Particularly, a set of lignin solutions in 90 vol% acetone (aq.) was prepared in the range of 120–600 mg L^−1^. Following that, each FL‐containing solution was mixed with a 75 µmol L^−1^ solution of DPPH in 90 vol% acetone (aq.) in a FL:DPPH = 1:39 (v/v) ratio. The obtained solutions were subjected to UV–vis spectroscopy using a Shimadzu UV‐2550 spectrophotometer by placing the solution into a 10 mm path length quartz cuvette. Absorbance at 515 nm was recorded immediately after mixing and again after 24 h, once equilibrium conditions were established. To account for DPPH self‐degradation in 90 vol% acetone (aq.), a blank solution (75 µmol L^−1^ DPPH solution in 90 vol% acetone (aq.) without lignin) was monitored over time. A full description of the experimental protocol is provided in the ESI.

### 2.6 Preparation of Regenerated Cellulose Films

The film preparation was performed according to Pang et al. Briefly, before preparing the lignocellulosic solutions (dopes), the ionic liquid [emim]Cl was pre‐melted in a water bath at 80°C. After melting, FL was added to [emim]Cl under continuous stirring to form a 1.5 wt% solution. Following a 30 min pre‐stirring period to allow for lignin dissolution, cellulose (3 wt%) was introduced. The mixtures were then heated at temperatures ranging from 80°C to 100°C under magnetic stirring until the cellulose was fully dissolved in the ionic liquid. The resulting solutions were then cast onto glass plates and immediately coagulated in water to form regenerated cellulose films. These films were thoroughly rinsed with distilled water and subsequently air‐dried. Prior to characterization, all films were conditioned at 25°C and 50% relative humidity to stabilize their moisture content.

### 2.7 UV–Vis

The optical properties of the lignocellulosic films were analyzed using an Agilent Cary 5000 UV–vis‐NIR Spectrophotometer across the 200–600 nm wavelength range. Film transparency was specifically evaluated at 550 nm. Additionally, the absorption percentage was measured within the UV‐B (280–320 nm) and UV‐A (320–380 nm) regions to assess the UV‐blocking properties of the prepared films.

## 3 Results and Discussion

The process underlying this study is illustrated in Figure 1. By means of green and flexible AqSO process, 3 pulp fractions were obtained by incorporating different *P*‐factors (500, 1000, 2000) at a constant temperature (*T* = 180°C) and *L*/*S* = 1. Following that, the resulting treated solids (pulp) were subjected to a set of various post‐HTT extractions (Figure 1) to isolate lignin/LCCs with differing structural characteristics. At the last stage, isolated lignin/LCCs were evaluated for their thermal (*T*_g_) and antioxidant properties, while their performance was examined through UV‐blocking efficiency (BE) in lignocellulosic film formulations.

### 3.1 The Effect of the Processing Conditions and Post‐HTT Sequential Fractionation on the Yield

FLs yields discussed in the current work were calculated based on dry wood (sawdust) mass using the formula (Equation (3)):

(3) $$\begin{array}{c} \text{FL yield}\left(\%\right)=\frac{m_{\mathrm{fl}}}{m_{\mathrm{sd}}\times \left(1-\mathrm{MC}\right)}\times 100 \end{array}$$

where *m*_fl_ is the dry yield of the selected fractionated lignin determined gravimetrically in g. Prior to measurement, the FLs were isolated by either solvent removal through rotary evaporation or precipitation (Section 2.1), followed by vacuum oven drying under P_2_O_5_ (*T* = 40°C, *p* = 0.1 mbar). *m*_sd_ is the mass of sawdust loaded into the reactor; MC is the moisture content of the sawdust expressed in fractional (decimal) form, determined prior to the experiments.

**FIGURE 2:**
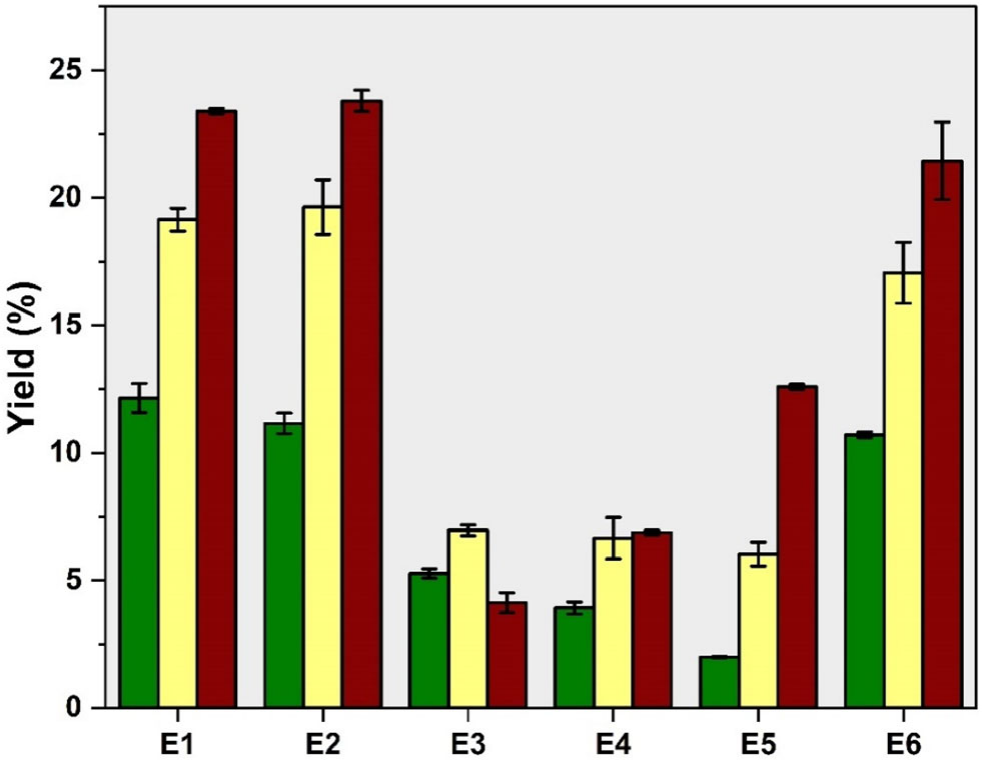
The effect of extraction conditions on the yield of FLs, where 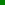 *P*‐factor = 500; 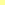 *P*‐factor = 1000; and 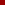 *P*‐factor = 2000. All experiments were performed twice.

Yields of FLs obtained under different extraction conditions clearly indicate that in all cases the highest yield corresponds to the highest *P*‐factor (2000), not depending on the used solvent (Figure 2). Notably, condition E3 yielded the highest extraction output at *P*‐factor = 1000. It might be associated with a high water‐content solvent (60 vol%), which may have restricted lignin extraction due to the formation of condensed structures under severe processing conditions evidenced in recent works [4, 11]. Importantly, condition E1 provided yields comparable to those of E2, and both are in good agreement with the results obtained in recently published work, where E1 extraction condition was employed. This outcome implies that sequential fractionation allows for to extraction of lignin that cannot be obtained using only 75 vol% acetone (aq.). In contrast, extractions E3 and E4 resulted in a ca. twofold decrease in yield under *P*‐factor = 500, almost threefold drop in yield at *P*‐factor = 1000, and 5.7 times less yield at *P*‐factor = 2000. It can be attributed to the solvent that was used during the E3 and E4 extractions. In both cases, EtOH‐based solutions with varying concentrations were employed. 40 vol% EtOH (aq.) (E3) is a highly diluted solvent that provides insufficient lignin extraction outcomes, while the subsequent extraction with 75 vol% EtOH (aq.) (E4) did not improve the yield. Interestingly, the follow‐up extraction with 75 vol% acetone (aq.) (E5) allowed to extract 3 times more lignin compared to E3 and ca. 2 times more compared to E4 at *P*‐factor = 2000, which ones again proves the formation of more condensed structures under higher *P*‐factors that are not readily soluble in EtOH but dissolve effectively in acetone‐based solvents. While the absence of a pronounced change in yields of E3, E4, and E5 extractions at *P*‐factor = 1000 can be detected, a drastic difference of over 62% between E3 and E5 at *P*‐factor = 500 is indicated, supporting the hypothesis that depolymerized lignin fragments at lower *P*‐factors can be solubilized by either solvent. Furthermore, extraction under condition E6 produced yields similar to E1 and E2 at *P*‐factor = 500, with only minor differences of 11% and 13% at *P*‐factor = 1000 and ca. 8.5% at *P*‐factor = 2000. These findings allow us to assume that FL yield is highly dependent on the post‐HTT solvent, with a prevailing effectiveness of E1, E2, and E6 that provide relatively high extraction yields over the *P*‐factor range. Overall, a post‐HTT extraction at *P*‐factor = 2000 provides equally high (20%–25%) lignin yields based on dry biomass regardless of the solvent used. Simultaneously, EtOH‐based solvents favor the extraction of depolymerized lignin fragments in higher and identical yields produced at *P*‐factor = 1000, marking E1, E2, and E6 as the most efficient post‐HTT extraction strategies to produce lignin/LCCs in high yields.

### 3.2 Structure of the Fractionated Lignins

**FIGURE 3:**
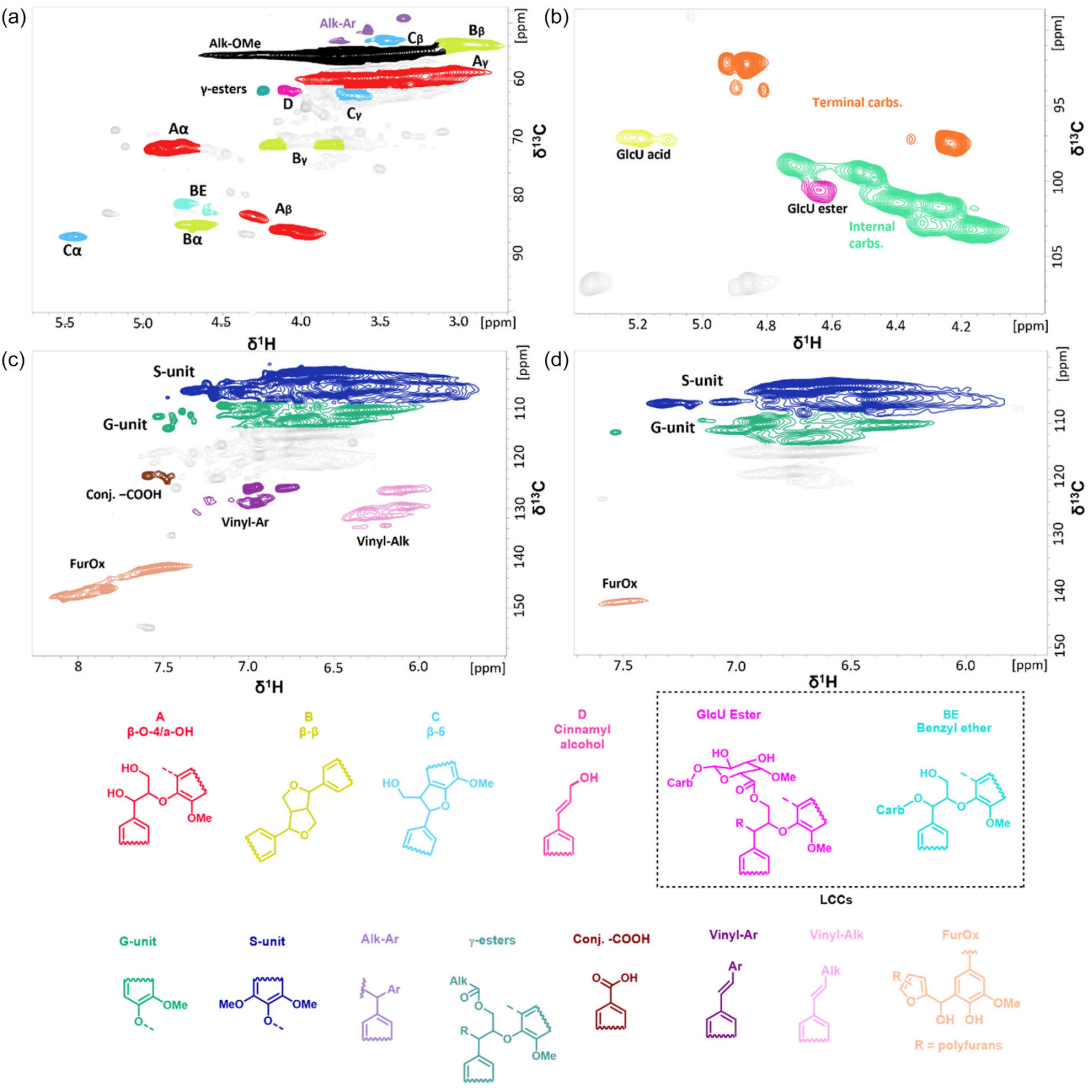
2D HSQC NMR spectra of fractionated lignin. The cross‐peaks of the areas of interest were assigned based on corresponding resonances. Distinct colors were used to highlight key moieties in the oxygenated aliphatic region (a), LCC occurrence (b), and aromatic regions of the 75 vol% acetone (aq.) fractionated lignins produced under the same conditions of *P*‐factor = 2000 (c,d). The presence of FurOx, Vinyl‐Alk, and Vinyl‐Ar structures is suggested based on earlier studies [8, 11].

**FIGURE 4:**
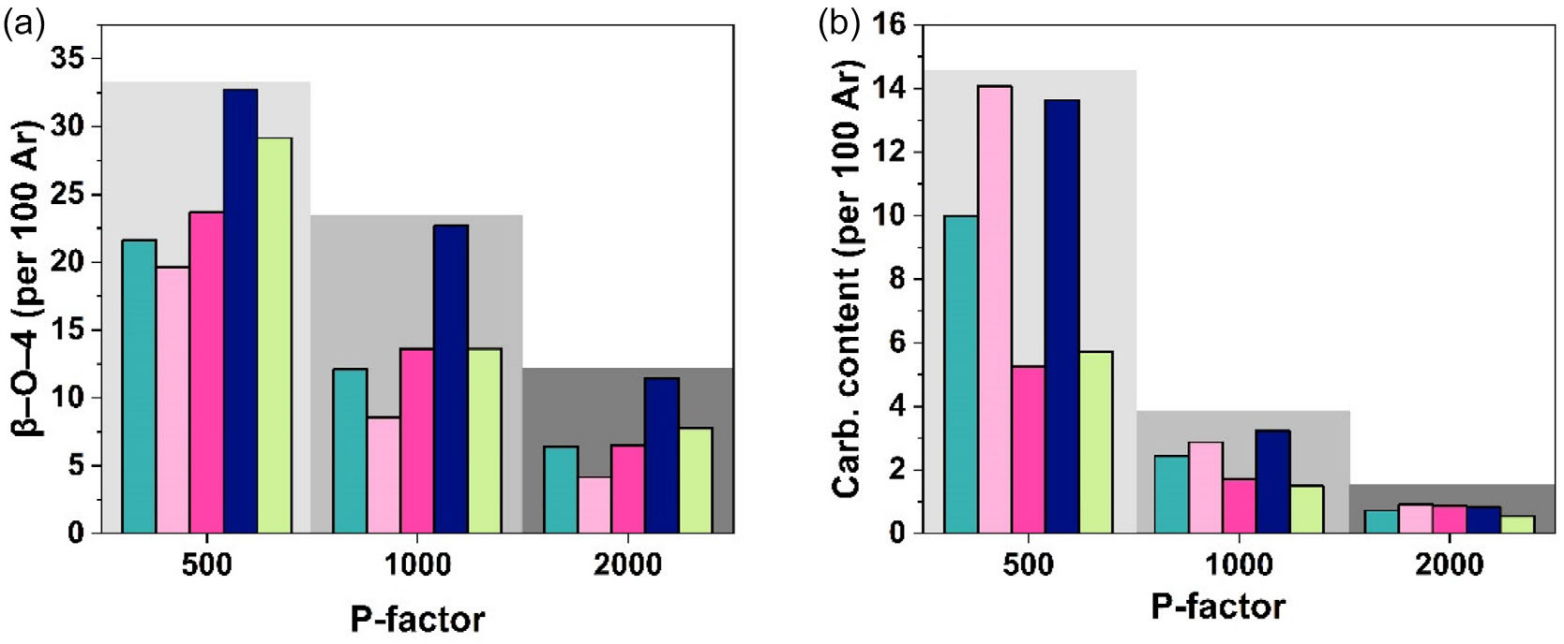
Semi‐quantitative distribution of different moieties in fractionated lignins yielded under various processing and extraction conditions: (a) *β*−O−4 and (b) carbohydrate content in regard to the severity of the process; where 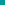 E1, 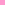 E3, 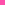 E4, 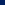 E5, 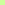 E6.

^1^H‐^13^C heteronuclear single quantum coherence spectroscopy (HSQC) analysis allowed to detection of the main structural features of the fractionated lignins after the post‐HTT fractionation (Figure 3). Consistent with previous reports [8, 11, 29], all lignins demonstrated a similar trend of *β*−O−4 linkages content (Figure 4a). While all FLs produced at *P*‐factor = 500 showed consistently high amount of *β*−O−4 linkages (structure A; Figure 3), sequential fractionation E5 provided a 51.4% increase in *β*−O−4 linkages preservation in comparison to E1, where only a direct 75 vol% acetone (aq.) extraction was employed (Figure 4a). E6 also yielded a *β*−O−4‐rich lignin, yet still possessing a 12% lower *β*−O−4 content than that of E5. Among the *P*‐factor = 500 selection, E3 provided a relatively low number of *β*−O−4 units of 19.6/100 Ar, which is comparable to that of E1. Interestingly, E5 resulted in 22.7/100 Ar at *P*‐factor = 1000, which is similar to the results obtained at *P*‐factor = 500. In contrast, other FLs obtained at *P*‐factor = 1000 demonstrated the amount of *β*−O−4 moieties in the range 8.6–13.6/100 Ar with E4 and E6 providing identical *β*−O−4 content of 13.6/100 Ar, that is 1.6 times higher than evidenced for E3. At *P*‐factor = 2000, all FLs presented a sharp drop in the number of *β*−O−4 units, which is in line with the previously reported works. Notably, E6 yielded lignin with only 9% difference in the amount *β*−O−4 moieties compared to E3 lignin produced at *P*‐factor = 1000. Across all processing conditions, E5 provided highest content of *β*−O−4 linkages in FLs, that might be associated with higher solubility of *β*−O−4 fractions in 75 vol% acetone (aq.). Additionally, in all cases, direct 75 vol% acetone (aq.), sequential 75 vol% EtOH (aq.), and alkaline extractions demonstrated similar efficiency in isolating *β*−O−4‐rich fractions, with a particularly higher (by ca. 35%) outcome of alkaline extraction at low severity (*P*‐factor = 500). This finding is highly relevant to conventional alkaline‐based pulping processes, offering a pathway to isolate *β*–O–4‐rich lignin for value‐added applications.

The presence of LCC linkages was allocated according to the characteristic cross‐peaks at *δ*_C_/*δ*_H_ 80–81/4.5–4.8 ppm for benzyl ether, *δ*_C_/*δ*_H_ 101.1/4.65 ppm for glucuronic ester, and at *δ*_C_/*δ*_H_ 100–102/4.7–5.1 ppm for phenyl glycoside types. The occurrence of the latter, however, has not been observed over the range of employed processing and post‐processing extraction conditions. As LCC linkages were semi‐quantified per 100 Ar or mol% in each FL, it allows to assume that the resulting LCC content per dry wood mass (Equation (4)) can be determined as:

(4) $$\begin{array}{c} \mathrm{LCC}\text{content}\left(\%\right)=\frac{m_{\mathrm{fl}}\times {\mathrm{LCC}}_{\mathrm{per}\;100\;\mathrm{Ar}}}{m_{\mathrm{sd}}\times \left(1-\mathrm{MC}\right)} \end{array}$$

where *m*_fl_ is the dry yield of the selected fractionated sample determined gravimetrically in g; ${\mathrm{LCC}}_{\mathrm{per}\;100\;\mathrm{Ar}}$ represents the LCC content determined by HSQC NMR per 100 Ar; $m_{\mathrm{sd}}\times (1-\mathrm{MC})$ is the dry sawdust mass used for the reaction in g. For instance, if 4 g of dry wood were used and the resulting FL yield was 10% (Equation (3)), this indicates that the isolated 10% lignin fraction contains the LC linkage content as determined by HSQC analysis.

E3 yielded the highest carbohydrate content of 14.1/100 Ar at *P*‐factor = 500, followed by E5 and E1 produced under the same severity (Figure 4b). All samples produced at high *P*‐factors resulted in an extremely low number of LC linkages regardless of the extraction used. Nonetheless, at low (500) to moderate (1000) *P*‐factors, E3 and E5 allowed to extract a similar number of carbohydrate‐containing lignin units, that is 40% higher than that of direct 75 vol% acetone (aq.) extraction at *P*‐factor = 500, while during the increase in severity, the difference between those becomes negligible. Intriguingly, 75 vol% EtOH (aq.) and alkaline extractions have been proven to be inefficient for targeted LCCs extraction over all *P*‐factors. The distinctive efficacy of E3 can be explained by the higher solubility of the carbohydrate‐containing fraction in water‐based solvents.

Vinyl–aryl (Vinyl–Ar) and vinyl–alkyl (Vinyl–Alk) type of structures are suggested to be present in the majority of the analyzed lignins (Figure 3c) at *δ*_C_/*δ*_H_ = 133.4–126.0/7.4–6.8 and 135.5–123.0/6.5–5.8 [8, 11, 42], respectively, with a particularly higher occurrence in samples produced at low (500) and moderate (1000) *P*‐factors. This is in line with the recently reported results [8, 11]. Additionally, based on recent assignments [8, 11], the peaks at *δ*_C_/*δ*_H_ = 149.5–137.2/8.25–7.20 ppm suggest the presence of furanic structures (FurOx) in the spectra of high severity lignins (*P*‐factor = 2000) (Figure 3c,d).

Taking everything into consideration, direct 75 vol% acetone (aq.), sequential 75 vol% EtOH (aq.), and alkaline extractions exhibited superior efficiency in isolating *β*−O−4‐rich lignin fractions, while alkaline extraction at *P*‐factor = 500 resulted in a 35% higher abundance of *β*−O−4 linkages. In turn, water‐based solvents (E3) allow for isolating LCC‐rich fractions with a notable decline in extraction efficiency once more concentrated solvent is applied.

### 3.3 MMD

The glass transition temperature (*T*_g_), average MM, and MMD of lignin are critical characteristics that affect the potential for various applications [43, 44].

Importantly, as MM and MMD were determined by size exclusion chromatography (SEC), calibrated using sulfonated polystyrene standards, the presence of carbohydrates significantly affects the volume and conformation of lignin in solution, since SEC separates molecules based on their hydrodynamic radius [43, 45, 46]. Consequently, in samples with comparable carbohydrate content, the observed MM and MMD primarily express the intrinsic molecular weight distribution of lignin. However, high levels of carbohydrates may distort the MMD measurements, potentially leading to erroneous estimations.

Samples with similar carbohydrate content (Table 2, entry 1–3, E3 and E5) produced under the same *P*‐factor in all cases demonstrate the high dependence of the lignin fraction on the extraction conditions. For instance, E5 extraction allowed to extraction of LCCs as the highest molecular weight was found for this sample produced at *P*‐factor = 1000 (Table 2, entry 2). Notably, alkaline extraction (E6) yielded the highest molecular mass sample at *P*‐factor = 2000, which implies that 1 wt% NaOH can be a suitable solvent for the extraction of highly condensed lignins produced under severe conditions, while EtOH‐based extractions represent the lowest molecular weight fraction, which cannot reflect the complex structure of lignin produced at high *P*‐factors. Generally, the MMD was found to vary systematically with increasing severity across all extraction conditions employed. It can be explained by the multiplicity of chemical reactions expressed as the presence of many different reactions occurring simultaneously or sequentially during the AqSO process [11], which yield low molecular lignins with increasing severity due to *β*−O−4 linkage cleavage, as well as the formation of condensed structures at *P*‐factors close to 2000 [4, 8].

**TABLE 2:** MM distribution of the fractionated lignin samples.

Entry	*P*‐factor	*M*_w_, g mol^−1^				
		E1	E3	E4	E5	E6
1	500	3900	1400	3600	11,600	5100
2	1000	7800	1100	2400	15,100	6500
3	2000	12,600	1100	2600	11,400	18,200

### 3.4 Glass Transition Temperature

Taking into consideration yields, resulting FL structure, and MMD, the following samples were chosen to be tested further for their physicochemical properties due to their potentially high relevance for value‐added applications and the possibility of industrial implementation (Table 3).

**TABLE 3:** Classification of the fractionated lignins used in the current work for physicochemical properties tests.

Entry	Sample	*P*‐factor	Extraction
1	S1	500	40 vol% EtOH (aq.) (E3)
2	S2	1000	75 vol% acetone (aq.) (E1, direct extraction)
3	S3		75 vol% EtOH (aq.) (E4)
4	S4		1 wt% NaOH (aq.) (E6)
5	S5	2000	75 vol% acetone (aq.), (E5, sequential fractionation)

*T*_g_ is a critical thermal parameter for biopolymers, expressing the shift from a stiff, glass‐like behavior to a flexible, amorphous state [47]. When the temperature exceeds *T*_g_, molecular motion increases, allowing the material to deform more easily. The ideal *T*_g_ depends on the performance requirements of the specific application.

**FIGURE 5:**
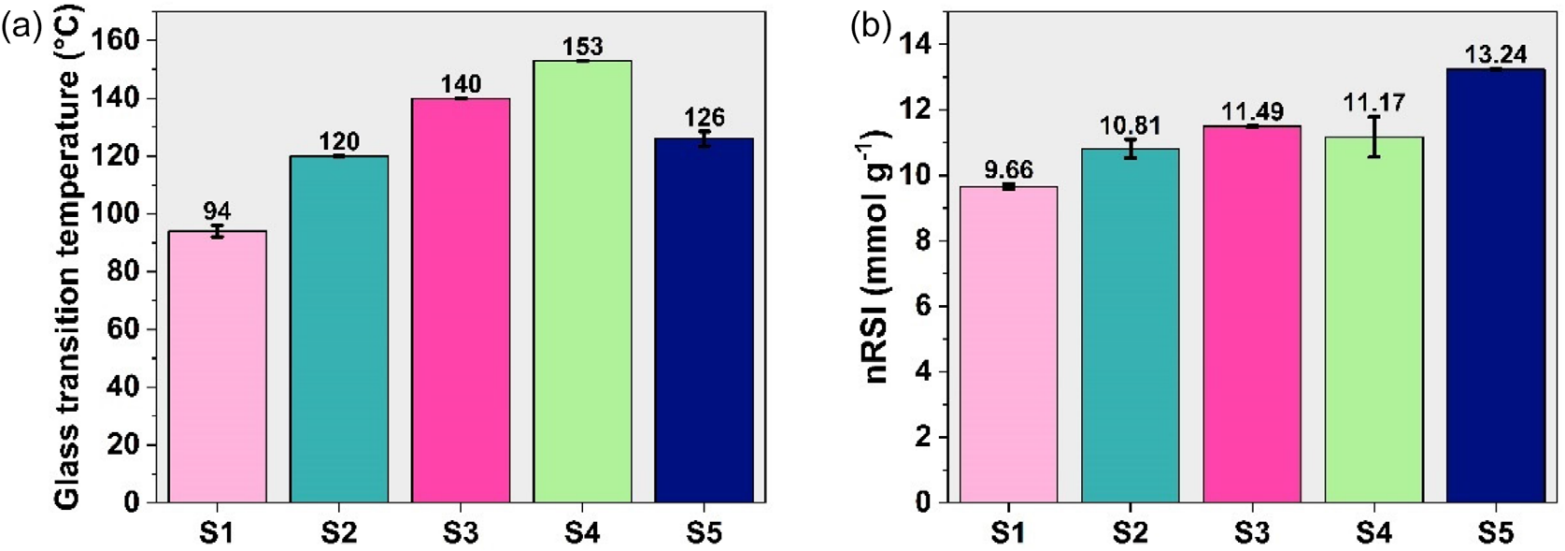
Physicochemical properties of fractionated lignins resulted from different processing and extraction conditions: (a) the distribution of the average glass transition temperatures across the fractionated samples; and (b) the magnitude of the antioxidant properties in fractionated lignins expressed through the nRSI.

Fractionated lignins showed a wide variability of the glass transition temperatures, ranging from 94°C to 153°C (Figure 5). Notably, 40 vol% EtOH (aq.) provided a low *T*_g_ fraction owing to the limited solubility of lignin fragments in water‐based solvents, simultaneously possessing a high abundance of *β*−O−4 linkages in the extracted fraction. Conversely, lignins yielded at *P*‐factor = 1000 demonstrated a progressive rise in the *T*_g_ values. S4 exhibited the maximum *T*_g_ observed across all tested lignins, similar to the value observed for Indulin AT in a recently published study [48]. These findings suggest that alkaline‐extracted lignins and technical lignins from the alkaline‐based Kraft pulping process have similar thermal characteristics in terms of *T*_g_. Although dialysis was not performed on S4, the NaOH‐extracted fraction was thoroughly and repeatedly washed with deionized water until neutral pH was achieved, implying effective removal of most residual salts and low‐molecular‐weight impurities. However, residual Na^+^ cations may participate in forming ionic interactions with phenolic and carboxylic groups in lignin, restricting segmental mobility and therefore increasing the glass transition onset point. In addition, S2 and S5 were found to have a similar thermal behavior to lignins produced in an earlier study [29]. Taking into account matching processing conditions, a reliable reproducibility of the AqSO biorefinery can be stated, which is of particular importance for producing tailor‐made lignins/LCCs. Remarkably, 75 vol% EtOH (aq.) outperformed 75 vol% acetone (aq.) in isolating lignins with high *T*_g_, compared to results reported previously [29]. However, S5 extracted with 75 vol% acetone (aq.), while produced at high *P*‐factor (*P*‐factor = 2000), did not result in a thermally rigid structure. By contrast, it demonstrated an 18% lower *T*_g_ in comparison to S4. This result allows us to assume that the extraction solvent plays a central role in isolating lignins with similar glass transition points, while the severity of the process does not provide a major impact on the resulting values.

### 3.5 Antioxidant Properties

Owing to their role in promoting thermal stability [49] and UV resistance [30], antioxidant properties are essential for ensuring the durability and functionality of materials over time. In light of this, all selected samples were tested for their antioxidant activity following the procedure suggested earlier (Figure 5b) [41]. In contrast to the tendency observed in *T*_g_ measurements, S5 demonstrated the highest antioxidant activity among all samples. Moreover, S5 showed a 22% higher antioxidant activity in comparison to S2, each isolated using the same solvent system (75 vol% acetone (aq.)). Unlike the tendency revealed for *T*_g_, it indicates that *P*‐factor contributes to a higher extent than the nature of the extraction solvent to the development of antioxidant properties, as the difference in the extraction conditions did not result in notable changes (Figure 5b; S2–S4). However, the residual Na^+^ present in the NaOH‐extracted S4 sample may reduce the accessibility of phenolic –OH groups, which are the main radical scavenging sites, therefore underestimating the antioxidant behavior of the given sample. Consistent with earlier findings [29], severe AqSO conditions allowed to production of lignins with enhanced antioxidant properties. Importantly, only a minor deviation of 3%–6% in antioxidant activity was observed for the samples produced under identical HTT conditions (*P*‐factor = 1000) regardless of the extraction solvent used. In relation to the outcomes presented in an earlier study [41], all fractionated lignins quantitatively demonstrated higher normalized radical scavenging index (nRSI) values than those determined for Indulin AT (9 mmol g^−1^), whereas S5 revealed antioxidant properties identical to those observed in Alcell. These outcomes confirm that *T*_g_ of AqSO lignins can easily be tuned according to the application requirements by varying the extraction conditions, while still exhibiting superior antioxidant properties without a notable dependance on the *P*‐factor applied.

### 3.6 ^31^P NMR

To advance the discussion on the process‐structure‐property correlation, ^31^P NMR analysis has been performed on selected samples (S1–S5). Alcell and Indulin AT (Table 4; entry 5 and 6, respectively) were added to the comparison as the representatives of the industrial pulping processes. Interestingly, S1, S2, and S4, with the highest content of –OH groups both phenolic and aliphatic (Table 4; entry 1,2, and 4), demonstrated less pronounced radical scavenging activities compared to those of S3 and S5 (Figure 5b). The latter, however, possesses 36% less –OH groups than S1 (Table 4) and 20% less –OH groups than S2. The reason for this might be the lower solubility of S5 in the common ^31^P NMR solvents, therefore providing data only on the soluble fraction rather than on the whole sample, whereas insoluble fractions might also contain available –OH groups for DPPH scavenging. Importantly, S1 exhibited a similar total –OH groups content (ca. 10% more) to Indulin AT with 12% less phenolic –OH groups and 43% more aliphatic –OH groups (Table 4; entry 1), resulting in 7% higher nRSI value (Figure 5b) in comparison to Indulin AT [41]. Furthermore, Alcell with 8% lower total –OH groups compared to Indulin AT, demonstrated an almost identical antioxidant activity (13.35 mmol g^−1^) to that of the highest demonstrated nRSI provided by S5 (13.24 mmol g^−1^, Figure 5b) with a similar aliphatic –OH content (1.55 mmol g^−1^ vs. 1.69 mmol g^−1^; Table 4, entries 5 and 6). Comparing two samples (S2 and S4) with close phenolic and aliphatic –OH content (Table 4; entries 2 and 4), it is evident that the resulting 3% different nRSI values (Figure 5b) cannot be attributed to either of those factors, while S3 with ca. 4% less aliphatic and 6% less phenolic –OH groups (Table 4; entry 3) exhibited 6% higher antioxidant activity in contrast to S2 and 3% more pronounced antioxidant activity as opposed to S4 (Figure 5b). That said, a drastically higher content of aliphatic –OH groups yields considerably lower antioxidant activity as evidenced by S1 and S3, with S1 possessing 67% more aliphatic –OH groups and similar phenolic –OH groups compared to S3 (Table 4; entry 1 and 3), and therefore resulting in 20% weaker antioxidant properties (Figure 5b). These findings, consistent with the previous results [41, 48], further confirm the detrimental effect of aliphatic −OH groups. A possible mechanism involves the inhibition of the scavenging activity of phenolic −OH groups via H‐bonding with aliphatic hydroxy groups. Overall, our findings corroborate that the antioxidant properties are not solely determined by the content of phenolic –OH groups but rather arise from the collective contribution of various lignin functionalities and structural peculiarities.

**TABLE 4:** Quantification of –OH groups in selectively fractionated lignins, Alcell, and Indulin AT performed by ^31^P NMR.

Entry	Label	–OH / –COOH groups in mmol g^−1^							Ref.
		–OH						–COOH	
		Aliph.	5‐Sub	Gnc	H	Phenolic	Total		
1	S1	3.64	2.40	0.65	0.16	3.21	6.85	0.32	Current paper
2	S2	2.26	2.49	0.62	0.16	3.27	5.54	0.33	
3	S3	2.18	2.30	0.61	0.16	3.07	5.25	0.34	
4	S4	2.28	2.42	0.63	0.18	3.23	5.51	0.40	
5	S5	1.55	2.16	0.53	0.18	2.87	4.42	0.29	
6	Alcell	1.69	2.73	1.02	0.25	4.00	5.69	0.37	[41]
7	Indulin AT	2.54	1.63	1.75	0.27	3.65	6.19	0.42	[41]

### 3.7 Lignocellulosic Films for EnhancedUV Protection

**FIGURE 6:**
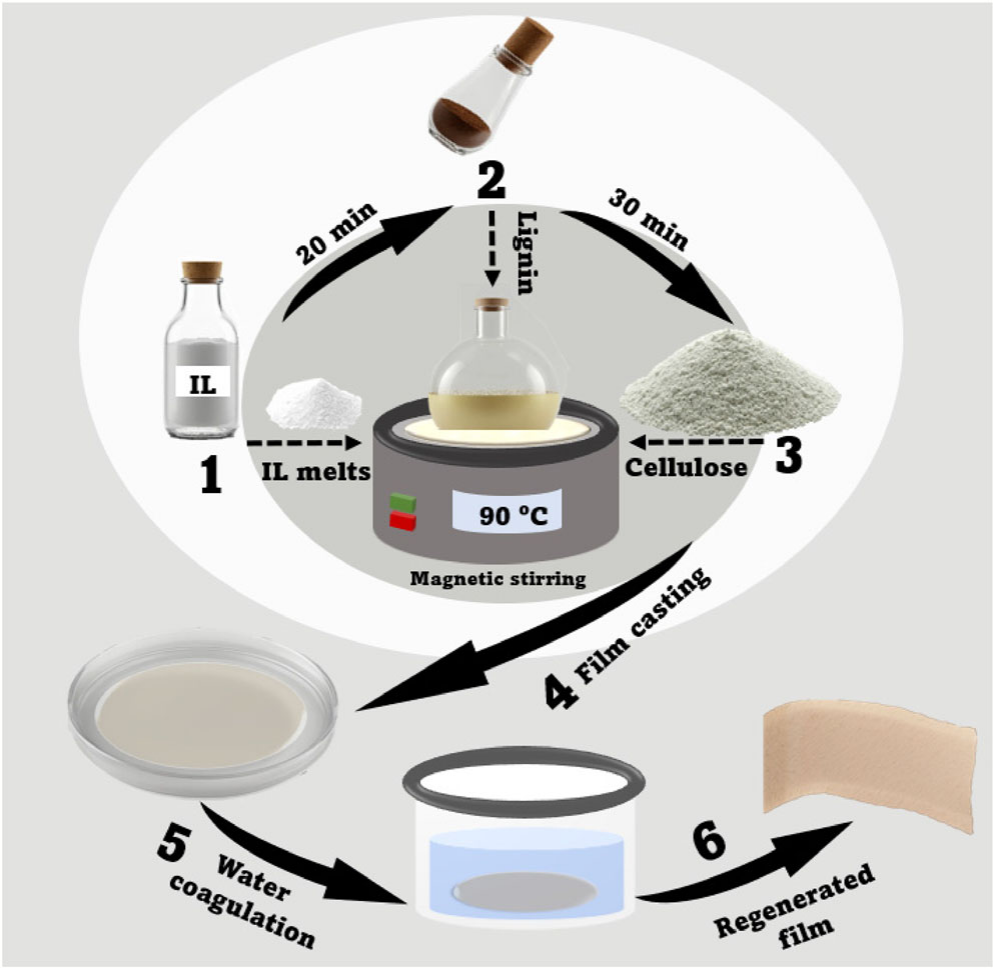
A schematic representation of the regenerated lignocellulosic film preparation.

Lignin, as a branched phenolic polymer, is chemically incompatible with linear carbohydrate biopolymers such as cellulose by simple mechanical mixing. To overcome this, Sadeghifar et al. [30] employed click chemistry to covalently bond functionally modified cellulose with azide groups and lignin containing propargyl groups. Although this approach demonstrated an exceptional UV‐blocking capacity of the resulting lignin‐containing cellulosic films, it involved additional chemical modifications, thus increasing both operational time and complexity of the process, which is neither feasible nor cost‐effective. In contrast, a solvent capable of dissolving both lignin and cellulose ([emim][Cl]) was employed in the current work, allowing for the preparation of a homogeneous solution that could be directly cast into uniform films (Figure 6). Table 5 summarizes other recently developed methods for the preparation of lignocellulosic films.

**TABLE 5:** Overview of lignocellulosic film preparation strategies reported in the literature.

Entry	Method	Preparation	Outcome	Ref.
1	Biomass extraction and subsequent crosslinking	The lignocellulosic residue of switchgrass biomass, extracted using alkaline (5 wt% KOH (aq.)) and bleaching treatments, solubilized in ZnCl_2_ solution, followed by crosslinking with calcium ions and casting onto a glass plate.	High tensile strength and biodegradability in soil, low water vapor permeability	[50]
2	Molecular remodeling strategy	Lignin with high phenolic –OHs and low M_w_ was separated from bamboo sawdust using a green deep eutectic solvent and combined with cellulose to prepare regenerated lignocellulosic films via coating and phase inversion. Lignin acted as an aromatic binder through hydrogen bonding with cellulose, while epichlorohydrin induced covalent cross‐linking.	Thermal stability, water resistance, UV‐shielding	[51]
3	CNF‐prehydrolysate lignin (PR) suspension	NaClO‐NaBr‐TEMPO oxidation to prepare TEMPO‐oxidized CNFs, followed by the formation of the CNF‐PR suspension in water with a subsequent casting onto a Petri dish.	UV‐blocking and antibacterial properties	[52]
4	Green immersing, drying, and hot‐pressing procedure	Mixture of microfibrillated cellulose (1 wt%) with lignin (0.5 g) in water is subjected to vacuum filtering, followed by immersing the semi‐dried mats in xylan solutions. After drying (60°C, 1 h; 110°C, 15 min, 0.1 MPa), the mats were hot‐pressed at 110°C under 10 MPa.	High thermostability, water and oxidation resistance	[53]
5	Biopolymers dissolution in [C_2_C_1_im][OAc]/DMSO	Lignin was pre‐dissolved in DMSO and then added with cellulose to the ionic liquid. The mixture was vortexed for 2 min and then heated to 70°C until complete dissolution.	High mechanical and UV‐blocking properties	[54]

To further elucidate the UV‐blocking capacity of lignocellulosic films, S3 and S5 were chosen for subsequent film fabrication based on the demonstrated superior antioxidant activity. In addition, Alcell, a commercially available organosolv lignin, was also incorporated into the comparison as a reference. The UV‐BE (%) was determined based on each film´s UV absorbance and transmittance values. The transmittance accounts for all light transmitted in the forward direction, excluding contributions from absorption and back scattering. At the first stage, the absorbance values across the targeted 200–600 nm wavelength range were converted to transmittance based on the Beer–Lambert law (Equations (5–7)) using the formula (Equation (8)) [55]:

(5) $$\begin{array}{c} A=\log_{10}\frac{I_{0}}{I} \end{array}$$

(6) $$\begin{array}{c} T=\frac{I}{I_{0}} \end{array}$$

(7) $$\begin{array}{c} A=-\log_{10}T \end{array}$$

(8) $$\begin{array}{c} T\left(\%\right)=10^{-A}\times 100 \end{array}$$

where *T* is a transmittance value determined for each absorbance value across the UV range; *A* is the absorbance value. Following that, an average transmittance across the implemented wavelength range was detected following Equation (9):

(9) $$\begin{array}{c} T_{\text{aver}}=\frac{1}{N}\sum_{i=1}^{N}T(\lambda_{i}) \end{array}$$

where *T*_aver_ is the average transmittance over the chosen wavelength range; *N* corresponds to the number of UV measurements across the UV range; $T(\lambda_{i})$ denotes the transmittance calculated using Equation (8) at each UV‐range wavelength. Finally, the resulting UV‐BE was determined by Equation (10):

(10) $$\begin{array}{c} \mathrm{BE}\left(\%\right)=100-T_{\text{aver}} \end{array}$$

**FIGURE 7:**
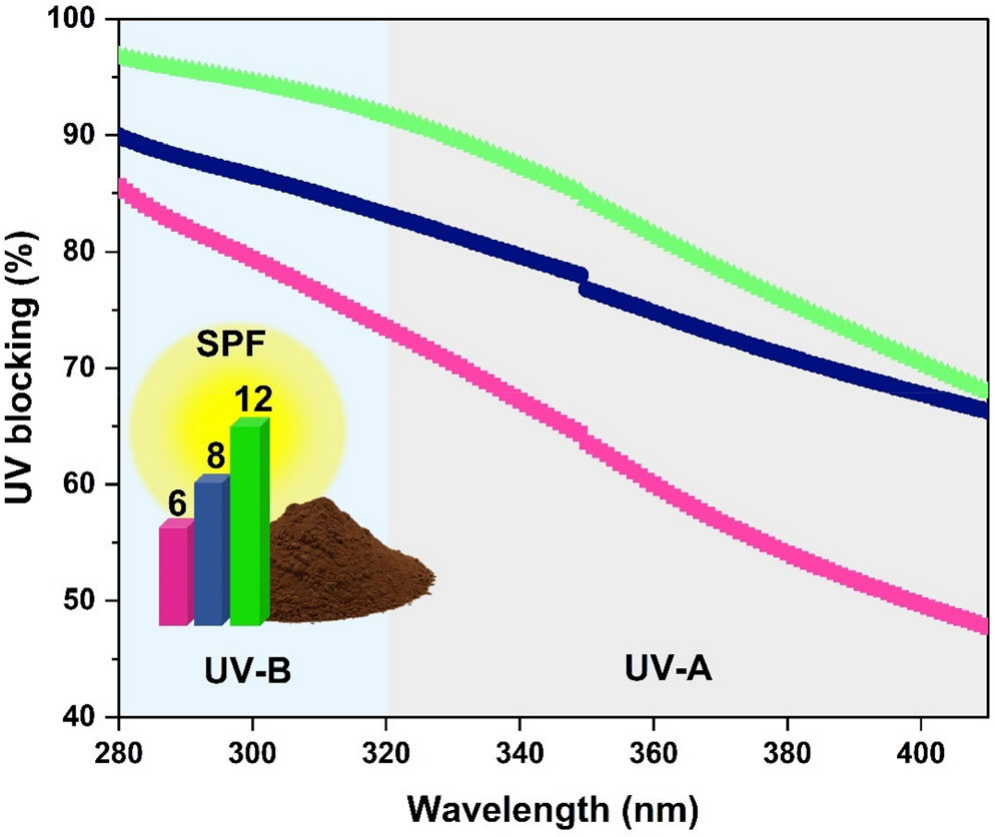
UV‐BE (%) and SPF values of the regenerated lignocellulosic films with 1.5 wt% of the selected fractionated lignins content added to each formulation and measured at UV‐B (280–320 nm) and UV‐A (320–400 nm) ranges (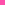 S3, 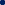 S5, and 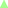 Alcell).

A considerable difference between the UV‐shielding capacities of the films was demonstrated (Figure 7). While S5 and Alcell showed more effective UV‐BE of 91% and 97%, respectively, S3 was proven to have only 87%. This variation is likely due to differences in processing conditions. For instance, S5 produced at *P*‐factor = 2000 demonstrated higher UV‐BE as a result of the extremely low carbohydrate content.

To quantify the UV‐protective potential of lignin, the sun protection factor (SPF), which is widely used in cosmetics and UV‐protective materials, was also determined based on the UV absorption of the fractionated lignins in the UVB spectrum (280–320 nm) range [56]. Generally, solar ultraviolet radiation comprises three spectral regions: UVC (200–280 nm), UVB (280–320 nm), and UVA (320–400 nm) [57]. Although UVC possesses the highest risk, it is almost completely filtered by the ozone layer. UVA, on the other hand, reaches the Earth's surface and produces long‐term cumulative effects associated with skin photoaging [58, 59]. Considering that UVB radiation exhibits a roughly 1000 times higher erythemogenic potential than UVA, the SPF predominantly quantifies the protective efficacy of a sunscreen formulation against UVB‐induced skin damage using the formula provided in Equation (11) [59]:

(11) $$\begin{array}{c} \mathrm{SPF}=\text{CF}\sum_{290}^{320}\mathrm{EE}(\lambda_{i})\times I\left(\lambda_{i}\right)\times A(\lambda_{i}) \end{array}$$

where CF is the correction factor that equals 10; $\mathrm{EE}(\lambda_{i})$ represents the erythemogenic effect of radiation at the selected wavelength $\lambda_{i};I(\lambda_{i})$ designates the solar intensity spectrum, representing the spectral distribution of solar irradiance as a function of wavelength; $A(\lambda_{i})$ is the absorbance at the chosen wavelength $\lambda_{i}$. The values expressing $\mathrm{EE}(\lambda_{i})$ and $I(\lambda_{i})$ are pre‐determined constants reported by Sayre et al. [60]

In the given context, Alcell showed a 2‐times higher SPF value of 12 in comparison to S3 (6), while maintaining a 1.5 times better sun protection than that of S5 (8) (Figure 7). It could also be associated with higher chromophore content in Alcell as a result of the pulping process that elevates its UV‐BE [17, 59]. The percentage of UV protection translates as: 100‐(100/SPF) [17], i.e., a material with an SPF = 12 (Alcell) can protect from 91.7% of the UVB light. In contrast, S3 is efficient in protecting up to 83.3% of UVB rays, while S5 provides 87.5% of protection. Conversely, choline citrate‐based ionic liquid stabilized gelatin–lignin films exhibited strong UV‐shielding (SPF up to 45) along with antimicrobial activity, while incorporating 0.5–1.5 wt% lignin into gelatin increased the SPF from 7 (pure gelatin) to 39–44.6 [61]. In addition, the transparency of the films was measured at *λ* = 550 nm through the films´ transmittance. The transmittance values represent the total forward‐directed transmitted light, excluding contributions from absorption and back‐scattering. A 1.5 wt% incorporation of Alcell into the formulation resulted in 59% transparency, with S5 showing similar results (62%), while S3 demonstrated the highest transparency for lignin‐containing film of 70%. These findings are in good agreement with recently reported results by Sadeghifar et al., where a minor lignin incorporation of 2 wt% yielded films with around 60% transparency at the same wavelength (*λ* = 550 nm), while a neat cellulose film displayed a transparency of about 95% [30], highlighting that the cellulose film alone cannot provide UV protection.

During the lignocellulosic films preparation, a major challenge using [emim][Cl] was the fast and considerable increase in viscosity induced by hydrogen bonding between the biopolymers and the solvent [54, 62, 63, 64], which hindered polymer dissolution at higher concentrations. Conversely, preliminary experiments showed that films produced from low‐concentration biopolymer solutions were extremely thin and fragile, making them difficult to handle. Once the optimal concentration was established, achieving a homogeneous lignin distribution remained challenging, mostly due to its structural heterogeneity resulting from processing conditions. To address this and ensure efficient lignin dissolution, the stirring time after lignin addition was adjusted accordingly to avoid lignin agglomeration in the solution. For instance, the dissolution of S3 was the most time‐efficient, while that of Alcell was the most extensive among all samples (Table 6).

**TABLE 6:** Dissolution behavior of lignin samples in [emim][Cl].

Sample	Dissolution time, h	Compatibility effectiveness
S3	0.5	high
S5	1	high
Alcell	2	Low‐to‐medium

Eventually, the addition of lignin at as low as 1.5 wt% was reasonable to achieve enhanced UV‐BE among all samples. The UV‐shielding capacity is heavily influenced by the processing conditions, highlighting the importance of choosing high *P*‐factors that allow for to production of universally relevant and broadly applicable lignins with low *T*_g_, superior antioxidant properties, and high UV‐BE. Moreover, the results demonstrate that selecting a suitable solvent for further post‐HTT extraction may result in a more pronounced UV‐blocking. The study confirmed that tailor‐made lignins can be broadly utilized across a wide range of applications where potent antioxidant and UV‐shielding capacities are concerned, while the resulting properties can be adjusted according to the desired performance already at the biomass processing stage. A selection of an appropriate solvent can enhance lignin performance even further, allowing for to isolation of fractions with targeted functional content that corresponds to the targeted application.

## 4 Conclusions

This study demonstrated the possibility of successful incorporation of lignin into the lignocellulosic films formulation, followed by the post‐HTT sequential fractionation, which allowed to isolation of lignin fractions with superior antioxidant and UV‐shielding properties, while providing an opportunity to design lignins with targeted thermal behavior as early as during the biomass processing. Importantly, although all tested lignins eventually demonstrated high UV‐blocking potential while being incorporated into lignocellulosic films, AqSO lignins (S3 and S5) exhibited improved compatibilization in the film‐forming mixture, while Alcell, as industrially produced lignin, demonstrated less facile solubilization. Simultaneously, 75 vol% EtOH (aq.) allowed to isolate lignins with higher *T*_g_ compared to those obtained from different extraction conditions, which implies that the extraction solvent plays a central role in producing lignins with a specifically targeted thermal behavior. In contrast, antioxidant properties were observed to be highly dependent on the processing conditions rather than on other parameters, yet providing potent antioxidant activity uninfluenced by solvent selection. These findings highlighted the opportunity of isolating lignins/LCCs engineered to reach a desired *T*_g_ with superior antioxidant properties. Moreover, the incorporation of FLs into the lignocellulosic film formulation at 1.5 wt% provided a UV protection of 87% to 97% depending on the severity of the processing conditions employed to produce the sample, whereas the SPFs values were ranging between 6 and 12, implying that when added in the correct concentration, lignin can provide an ultimate sun protection comparable to those of commercial UV‐filters. That said, as a renewable, nonhazardous, and environmentally friendly option, lignin provides a safe biobased alternative for UV protection in thermoplastics, cosmetics, and biomedical applications.

## Supporting Information

Additional supporting information can be found online in the Supporting Information section. **Supporting Fig. S1:** A visual representation of the yields of the solid residues obtained after chosen post‐HTT extractions, where 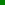 *P*‐factor = 500; 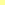 *P*‐factor = 1000; and 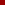 *P*‐factor = 2000. All experiments were performed twice. **Supporting Fig. S2:** Representation of the determination of the EC_50_ value for FL after 24 h. The intercept (x) of the IP = 50% and the fitting curve provide the concentration of lignin needed to scavenge 50% of the initially present radicals (*y*‐axis). **Supporting Fig. S3:** Replicated DSC thermograms of different FL samples. Glass transition temperatures are represented as average. **Supporting Fig. S4:** ^31^P spectra of the lignin samples employed in properties evaluation. **Supporting Fig. S5:** Absorbance of the tested lignocellulosic films with 1.5wt% of lignin as a function of the wavelength. **Supporting Table S1:** Average yields of fractionated lignins. **Supporting Table S2:** Comparison of the composition of the different solid residues after extractions.

## Author Contributions

**Daryna Diment:** investigation, methodology, conceptualization, writing – original draft preparation. **MiJung Cho:** investigation, methodology. **Michael Hummel:** conceptualization, supervision, writing – reviewing and editing, and funding acquisition. **Davide Rigo:** conceptualization, supervision, writing – reviewing and editing.

## Funding

This study was supported by Academy of Finland (341586, 316601, 341596/2021, 318890, 318891) and Business Finland (43674/31/2020).

## Conflicts of Interest

The authors declare no conflicts of interest.

## Supporting information

Supplementary Material

## Data Availability

The data that support the findings of this study are available from the corresponding author upon reasonable request.
