# Supplementary material for: Fractionation of AquaSolv Omni Biorefinery Lignins and Their Application in Antioxidant and Ultraviolet‐Protective Films
Source: ChemSusChem. 2026 Feb 18;19(4):e202501985. doi: 10.1002/cssc.202501985 (PMC12916230; doi:10.1002/cssc.202501985)
Supplement: Supplementary file 1 — Supplementary Material [file CSSC-19-e202501985-s001.pdf]

Fractionation of AqSO biorefinery lignins and their application in antioxidant and UV-protective films

Daryna Diment,[a] MiJung Cho,[a] Davide Rigo,[a] Michael Hummel [a,*]

[a] *Department of Bioproducts and Biosystems, School of Chemical Engineering, Aalto University, Vuorimiehentie 1, 02150 Espoo, Finland*

*Corresponding author: michael.hummel@aalto.fi

Table of contents

1. Yields of solid residue (treated biomass after extraction) 2
2. The composition (mass %) of the solid residues after extractions employed in the current study..3
3. Antioxidant activity (nRSI) 4
4. Glass transition temperature 6
5. ^{31}P NMR spectra 7
6. UV measurment plots 8

1. Yields of solid residue (treated biomass after extraction)

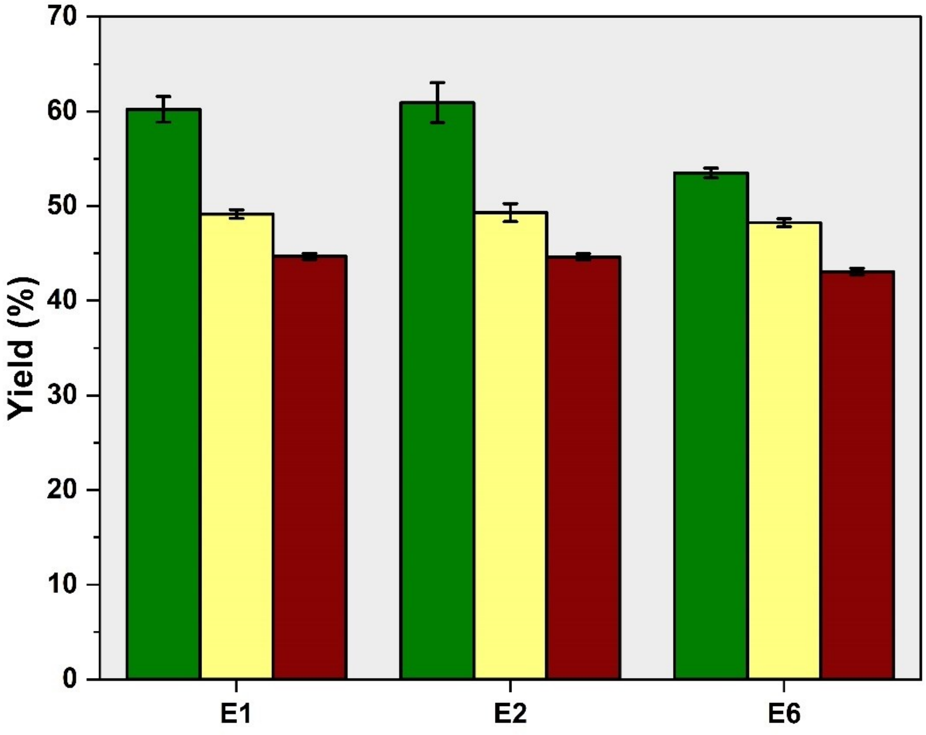

Figure S1. A visual representation of the yields of the solid residues obtained after chosen post-HTT extractions, where ■ P-factor = 500; ■ P-factor = 1000; and ■ P-factor = 2000. All experiments were performed twice.

Table S1. Average yields of fractionated lignins

**Fractionated lignin yields per initial lignin content, %**						
**P-factor**	**E1**	**E2**	**E3**	**E4**	**E5**	**E6**
**500**	55.18±2.59	50.68±1.84	23.89±0.83	17.82±1.05	8.46±0.46	48.63±1.10
**1000**	86.96±2.05	89.23±4.86	31.63±0.98	30.21±3.70	27.40±2.14	77.49±5.41
**2000**	106.28±0.52	107.14±1.89	18.72±8.10	31.24±4.95	57.18±2.26	97.40±6.90
**Fractionated lignin yields per dry wood, %**						
**P-factor**	**E1**	**E2**	**E3**	**E4**	**E5**	**E6**
**500**	12.15±0.57	11.16±0.41	5.26±0.19	3.92±0.41	1.97±0.23	10.71±0.01
**1000**	19.15±0.45	19.65±1.07	6.97±0.45	6.65±0.22	6.03±0.82	17.07±0.47
**2000**	23.41±0.12	23.59±0.21	4.12±0.40	6.88±0.09	12.59±0.10	21.45±1.52

2. The composition (mass %) of the solid residues after extractions employed in the current study

Table S2. Comparison of the composition of the different solid residues after extractions

Entry	Sample	Glc	Xyl	Man	Total carbs	AIL	ASL	Total lignin	Sum
1	Extracted birch (initial material)	41.15	20.36	1.96	64.46	17.74	4.29	22.02	86.49
	**P-factor**	**Composition after HTT**							
2	500	53.08	8.98	0.96	63.18	31.35	2.45	33.80	96.99
3	1000	51.97	5.12	0.54	57.64	32.61	1.38	34.00	91.63
4	2000	52.10	2.82	0.50	55.42	37.95	1.21	39.16	94.58
	**P-factor-Extraction**	**Composition after extraction**							
5	500-E1	61.50	9.18	0.00	70.79	16.83	1.93	18.76	89.55
6	500-E2	61.26	0.88	0.55	62.83	18.07	3.34	21.41	84.23
7	500-E6	65.65	8.48	1.24	75.51	17.85	3.21	21.06	96.56
8	1000-E1	71.90	6.87	0.76	79.53	11.24	0.68	11.92	91.45
9	1000-E2	72.66	6.89	0.75	80.30	11.43	0.76	12.19	92.49
10	1000-E6	72.27	6.70	0.75	79.72	10.63	0.68	11.31	91.03
11	2000-E1	85.33	4.61	0.36	90.31	8.82	5.12	13.94	104.25
12	2000-E2	86.77	4.73	0.37	91.88	9.37	5.06	14.44	106.32
13	2000-E6	88.55	4.87	0.38	93.80	7.51	4.09	11.60	105.40

A significant observation is that the total composition (%) exceeded the expected 100% level at P-factor = 2000 after each extraction due to the possible formation of pseudo-lignin which refers to carbohydrate-derived compounds (*e.g.*, humins or polyfurans formed from furfural, a xylan degradation product) that interfere with Klason lignin analysis by contributing to the erroneous estimation of wood composition.[1,2] This also applies to Table S1.

3. Antioxidant activity (nRSI)

The antioxidant properties of the FLs were quantified using the normalized radical scavenging index (nRSI), which was calculated following the procedure reported earlier.[3]

Briefly, the effect of the solvent system on DPPH self-degradation was evaluated by subtracting the absorbance of DPPH at a final time point (24 h) from the initial absorbance of DPPH (at 0 time point) measured through UV-Vis. It provides the correction factor ($\mathrm{cDPPH}_{abs}, Eq.S1$) for further calculations.

$$\mathrm{cDPPH}_{abs} = \mathrm{DPPH}_0(\mathrm{absorbance}_{\mathrm{t=0}}) - \mathrm{DPPH}_{24}\,(\mathrm{absorbance}_{\mathrm{t=24}}) \qquad (S1)$$

where DPPH_{24} is the absorbance at the time point of interest (t = 24 h) and DPPH_0 is the absorbance of DPPH at the initial time point. Following that, this correction factor (cDPPH_{abs}) was added to the absorbance values of the experimental lignin-containing solutions (A_{st}) monitored over 24 h (eq. S2):

$$A_{corr} = A_{st} + \mathrm{cDPPH}_{abs} \qquad (S2)$$

where A_{corr} is the corrected absorbance of the tested lignin-DPPH solutions. As A_{st} includes the absorbance of both DPPH and lignin (lignin-DPPH solution) after 24 h, A_{corr} is further used for the calculation of antioxidant activity of FLs used in this study.

The percentage of the scavenged DPPH radicals is expressed through inhibition percentage (IP) calculated by Eq. S3:

$$\mathrm{IP}(\%) = \frac{(\mathrm{DPPH}_0 + A_l) - A_{corr}}{(\mathrm{DPPH}_0 + A_l)} \times 100 \qquad (S3)$$

where A_l is the absorbance of FL solutions (without DPPH) at various defined concentrations (0.12-0.6 mg mL^{-1}) determined for each solution before adding the DPPH solution.

Subsequently, the effective concentration (EC_{50}) of FL at which 50 % of initially introduced DPPH radicals were neutralized, was found by plotting IP as a function of FL concentration in the tested lignin-DPPH solution as demonstrated in Figure S1.

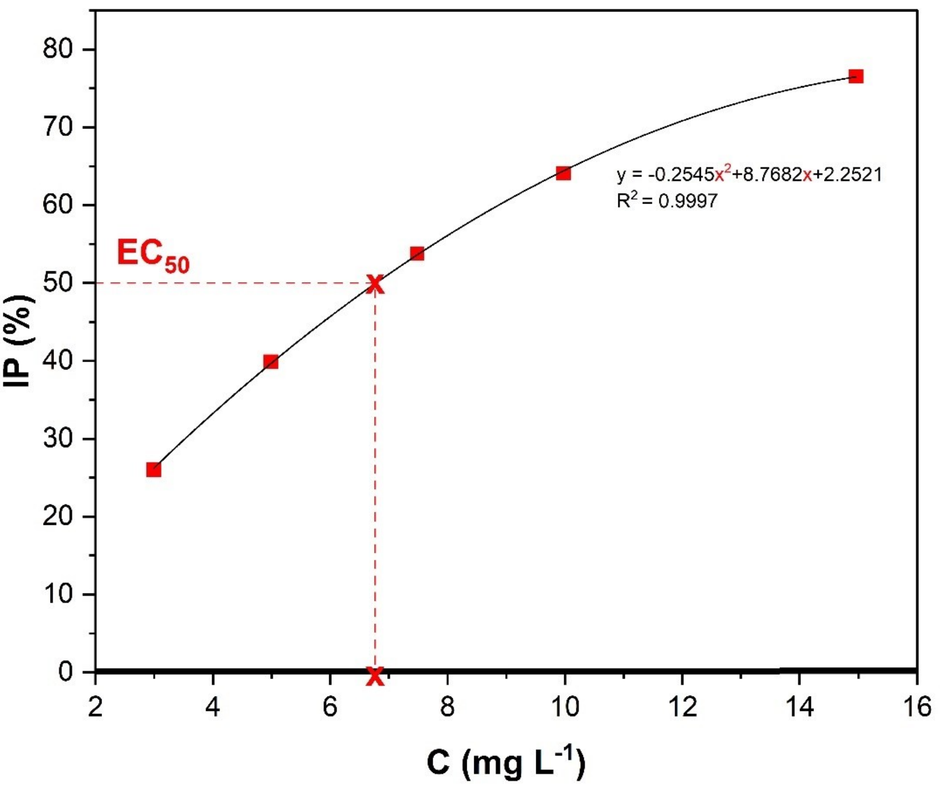

Figure S2. Representation of the determination of the EC_{50} value for FL after 24 hrs. The intercept (x) of the IP = 50 % and the fitting curve provide the concentration of lignin needed to scavenge 50 % of the initially present radicals (y-axis)

To minimize errors in estimating the antioxidant potential of lignin, the EC_{50} value is normalized by DPPH concentration (nEC_{50}, Eq. S4). At the last stage, the inversed value of nEC_{50} is referred as a normalized radical scavenging index (nRSI) (Eq.S5).

$$\mathrm{nEC}_{50} = \frac{\mathrm{EC}_{50}}{[\mathrm{DPPH}]} \quad (S4)$$

$$\mathrm{nRSI} = \frac{1}{\mathrm{nEC}_{50}} \quad (S5)$$

where EC_{50} is the effective concentration of lignin at which 50 % of DPPH radicals were scavenged (Figure S1), $[\mathrm{DPPH}]$ is the selected concentration of DPPH in the solution presented in mmol L^{-1}, nEC_{50} is the normalized effective concentration, whereas nRSI is a quantitative depiction of the antioxidant properties expressed as mmol of DPPH quenched by 1 g of lignin (mmol g^{-1}).

4. Glass transition temperature (T_g) measurements

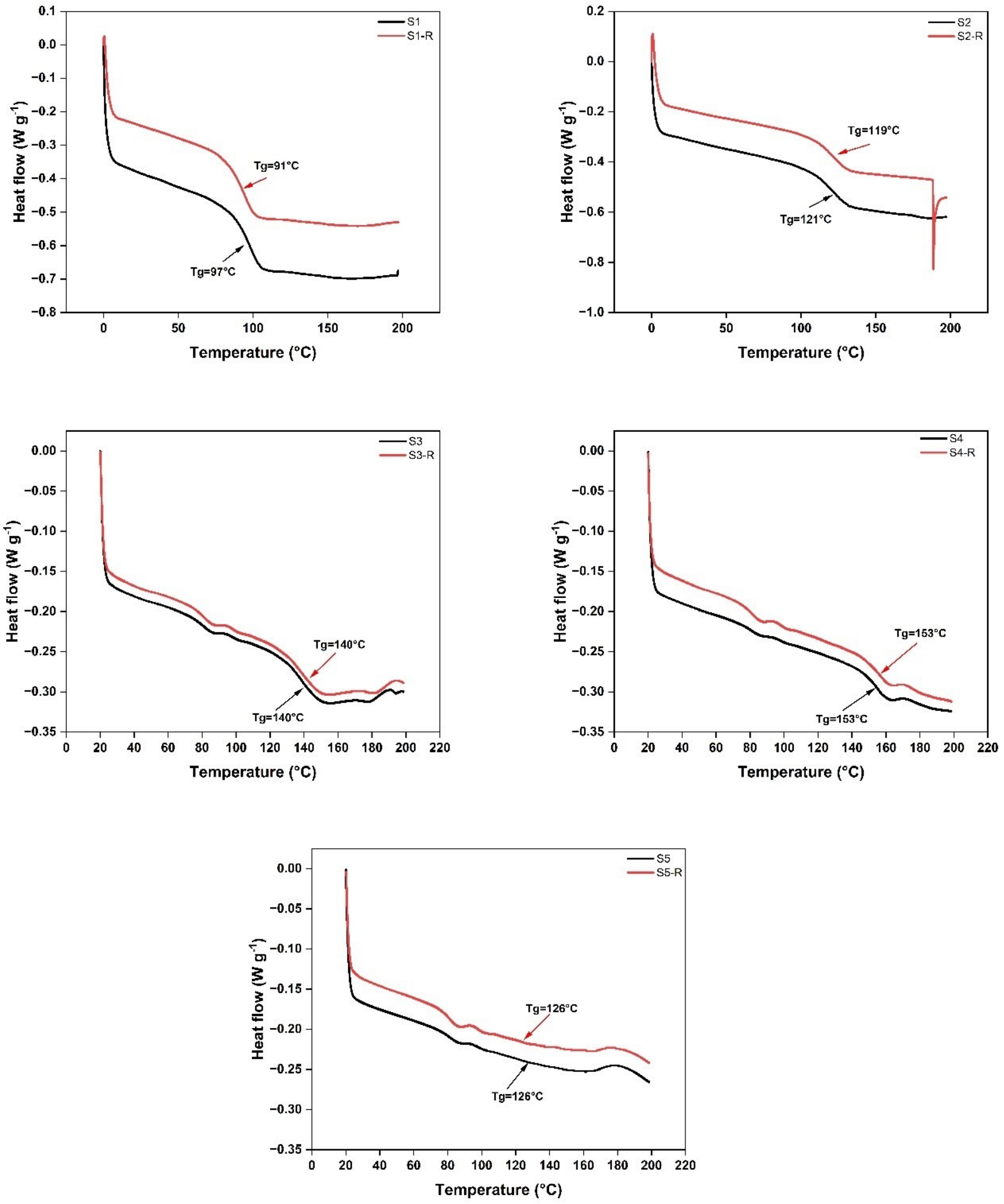

Figure S3. Replicated DSC thermograms of different FL samples. Glass transition temperatures are represented as average.

5. 31P NMR spectra

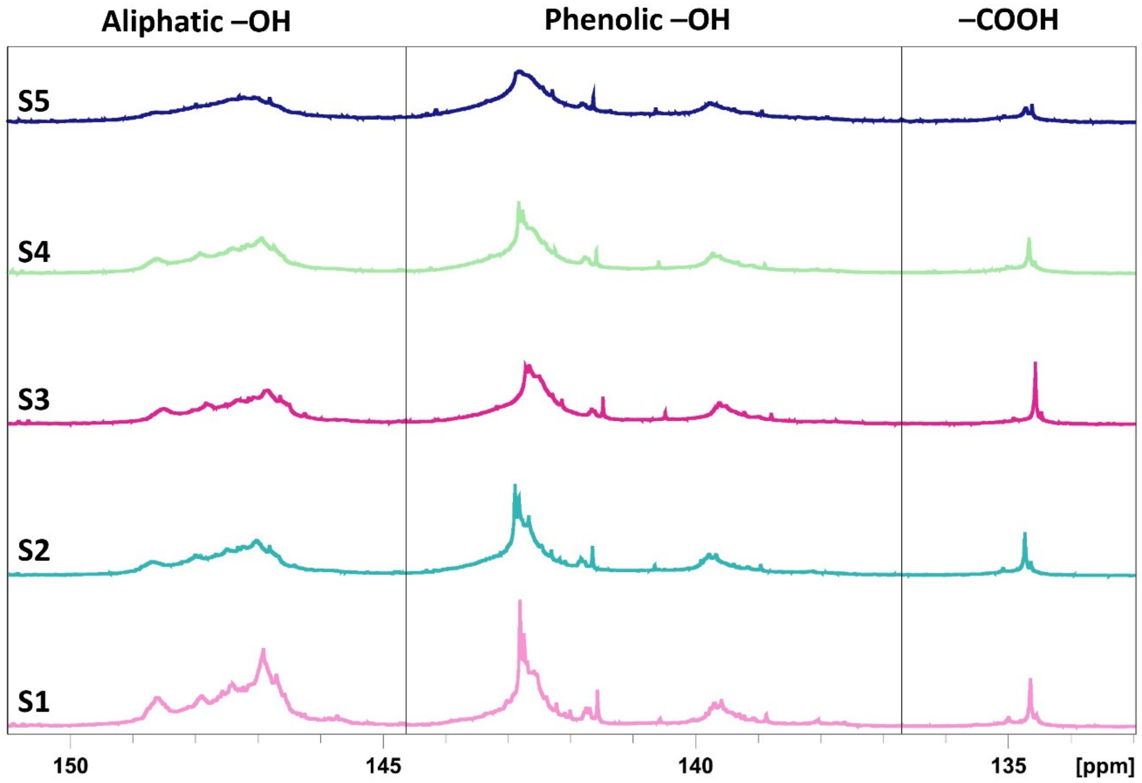

Figure S4. ^{31}P spectra of the lignin samples employed in properties evaluation.

6. UV measurment plots

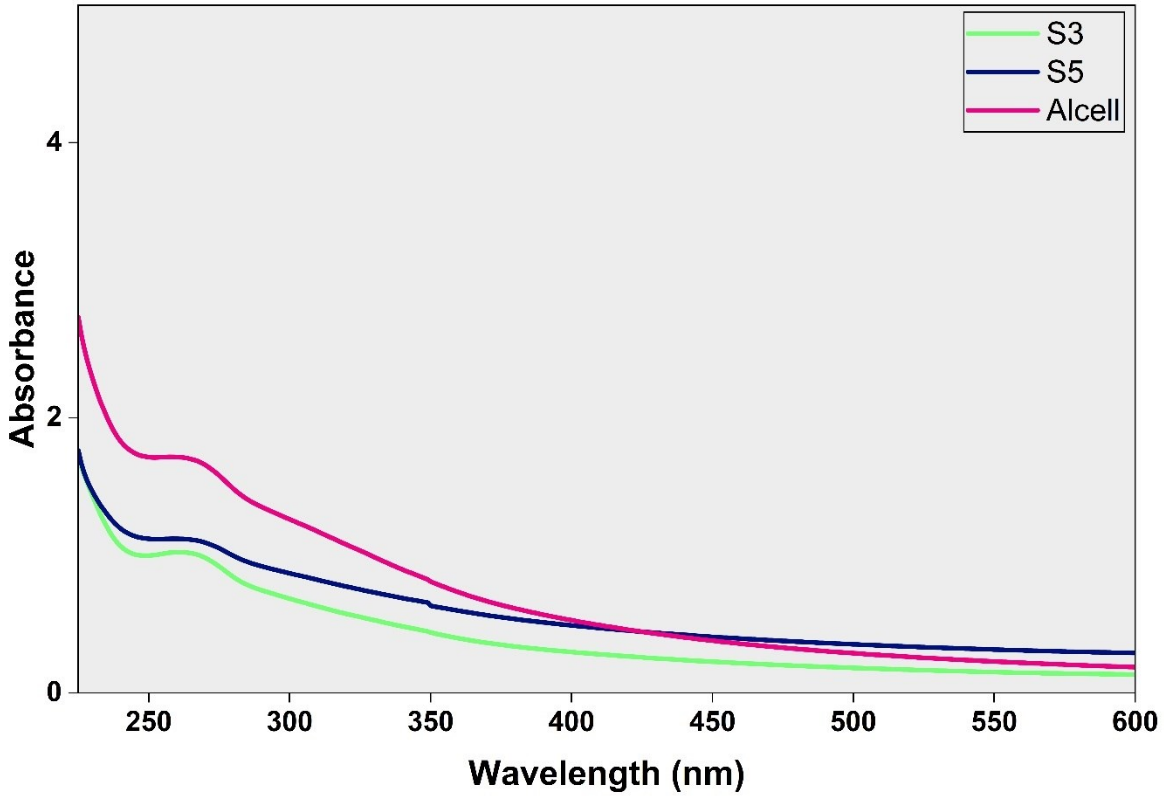

Figure S5. Absorbance of the tested lignocellulosic films with 1.5wt% of lignin as a function of the wavelength.

References

1 D. Tarasov, P. Schlee, A. Pranovich, A. Moreno, L. Wang, D. Rigo, M. H. Sipponen, C. Xu and M. Balakshin, *Green Chemistry*, 2022, **24**, 6639–6656.

2 A. Sluiter, B. Hames, R. Ruiz, C. Scarlata, J. Sluiter, D. Templeton, D. Crocker *Lab. Anal. Proced*, 2008, **1617**(1), 1-16.

3 D. Diment, O. Musl, M. Balakshin and D. Rigo, *ChemSusChem*, 2025, e202402383.
